# Industrial prospects on regulatory gaps and barriers in pharmaceutical exports and their counteraction: Local experiential with global implication

**DOI:** 10.1371/journal.pone.0305989

**Published:** 2024-07-19

**Authors:** Zobia Mubarak, Nasir Abbas, Furqan Khurshid Hashmi, Hina Shahbaz, Nadeem Irfan Bukhari

**Affiliations:** 1 Punjab University College of Pharmacy, University of the Punjab, Lahore, Pakistan; 2 Faculty of Pharmaceutical Sciences, Qarshi University, Lahore, Pakistan; Bolton Clarke Research Institute, AUSTRALIA

## Abstract

**Background:**

The pharmaceutical sector in Pakistan has grown over a period with export potential, however, there are certain barriers in the framework that regulate the growth and export of domestically manufactured pharmaceuticals. The purpose of this study was to highlight the current challenges that hinder the export of pharmaceuticals, especially to the countries with stringent regulatory authorities (SRA), as perceived by the domestic pharmaceutical industry experts, and to highlight the facilitators that may help to resolve the identified challenges.

**Methods:**

In a qualitative study, the data were collected from the consented experts from the pharmaceutical industries in Lahore, Karachi, Peshawar, and Quetta. Industrial experts with a minimum of 10 years of experience and who were serving at managerial levels or above were recruited through purposive sampling. The semi-structured interviews were conducted for the collection of data from industrial experts. Thematic content analysis was applied to conclude the data.

**Results:**

Data analysis generated 4 themes and 16 codes. The export of pharmaceuticals, despite having greater potential was regarded as poor, which was attributed to the following: (a) inadequate industrial research and development, particularly on new molecules (b) non-compliance with the cGMP standards, (c) absence of high-tech equipment, (d) unwillingness of the pharmaceutical companies for bioequivalence studies on their generics, (e) unavailability of locally manufactured active pharmaceutical ingredients, (f) disruption in the supply of imported raw material, (g) poor international market perception about local pharmaceutical products and (h) lack of support from regulatory in process expedition. The respondents also suggested the measures for overcoming the above challenges to boost the export of domestic pharmaceuticals and expand their international market share in countries with SRA.

**Conclusion:**

Export from Pakistan to the SRA countries can be enhanced with mandatory bioequivalence studies during generic registration. The pharmaceuticals export could effectively contribute to the national economy.

## 1 Introduction

A causal correlation exists between the health of a population and the economic progress of a nation [1]. One of the critical facets of public health pertains to the availability of efficacious medications, which constitute a considerable portion of healthcare outlays [2]. The pharmaceutical industry plays the above role in the provision of medicines to the public against sustainable business activity [3].

At the time of the establishment of Pakistan (1947), no pharmaceutical manufacturing facilities existed. By the 1960s, a few local manufacturers emerged, but the majority of medications were still imported, and there were no regulations to oversee the industry until 1967. Multinational companies (MNCs) also started entering the market [4]. Since then, the industrial landscape has undergone significant transformation. Though a consensus is to establish, Intercontinental Medical Statistics (IMS) Health Quintiles and by the way of (VIA) (IQVIA), estimates the existence of 759 active manufacturers [5]. Whereas, the Drug Regulatory Authority of Pakistan (DRAP), the governing body for the pharmaceutical sector, lists 637 registered firms in September 2018. Nonetheless, industry experts generally agree that the number of firms exceeds 700. The pharmaceutical companies are mainly located in Punjab and Karachi, Sindh. The domestic pharmaceutical manufacturing industry is characterized by a dichotomy between MNCs and domestic firms. Before 2010, 38 MNCs were operating in the sector, accounting for 60% of the market share. Nonetheless, in recent years, the MNCs after having gradually withdrawn, have decreased to 22 which resulted in a shrinkage of market share to 40% [6] and a shift in the balance of power, enabling local manufacturers to gain ground in the industry. The government exercises strict control over the country’s pharmaceutical industry through the DRAP, which oversees the manufacturing facilities, registration of new medicines, and setting the maximum retail price (MRP) for all pharmaceutical products sold in the country [7].

Pakistan’s healthcare market is primarily funded out-of-pocket by the public relying on personal expenditures to cover healthcare expenses. The government provides only free or low-cost medical treatment through public hospitals, clinics, or health initiatives named as *Sehat* (health) *Sahulat* (facility/service) program [8]. The private health insurance sector is expanding, offering hospitalization coverage to the public. Public-Private Partnerships with pharmaceutical companies collaborating with the government and non-governmental organizations are emerging to increase public access to medicines [9].

The domestic pharmaceutical industry, despite its substantial size, has not demonstrated the expected growth and dynamism of a thriving industry and has remained uncompetitive on the global stage, with minimal exports [10]. Further, the domestic pharmaceutical sector has not experienced the same trending upward level of growth and vibrancy as other industries are [5]. Domestic pharmaceutical exports have been valued at merely around US$200 million, tremendously lower than the India’s pharmaceutical exports worth US$14 billion in 2015 and even Jordan’s US$800 million exports, despite having a much smaller population of only 9 million. Moreover, India has 201 US-FDA-certified plants which is an impressive number, while Jordan has 4 certified plants, enabling the above both countries to export to the US, accounting for 60% of the global market. In contrast, Pakistan’s low exports and lack of US-FDA-approved firms, the international standard requirement demonstrate the country’s inadequate competitiveness and substandard products [11].

Literature establishes local and international issues that prevent domestic firms from exporting to markets with stringent regulatory authority (SRA), controlled by the USA, European Union, Japan, Australia, and Russia [12]. The market share, within the industry has become top-heavy, with the top 100 firms out of approximately 759 (IQVIA figure) controlling 97% of the market, leaving only 3% of the market for the remaining 650 firms [10]. This skewed structure, coupled with the lack of competitiveness, indicates the sector’s inability to positively impact national economic growth and public health [12]. The absence of backward linkages, i.e., to the suppliers is a reason for the lack of a domestic pharmaceutical industry, with 95% of raw materials being imported [5]. The industry-recognized the Section 12 of the Drug Act (Power to fix maximum prices of drugs) is the major obstacle in the progress of the export of pharmaceutical products [13]. A past study revealed the counterfeit medicine, pricing controversies, affordability of the medicine, lack of real research and development (R&D) initiatives, and unethical marketing (bribing/cash incentives/kickbacks to the doctors) as the challenges faced by the local pharmaceutical sector [14].

A no-objection certificate for export and a certificate of current good manufacturing practices (cGMP) needed from DRAP to export, along with bioequivalence certification from any accredited agency are considered as barriers. Yet, local facilities for testing and bioequivalence studies are scarce, though the reasons for the above lacking have not been reported [12].

The scarcity of WHO-, FDA- or EMA-accredited quality control laboratories or internationally known facilities offering FDA certification for export purposes are also irrevocable constraints [12]. Further, the import of raw materials, lack of industrial R&D, internationally-approved laboratory facilities, and accessibility to international facilities are the barriers that impede the pharmaceutical industrial growth [15].

There are certain issues related to export destinations. The industrial growth for exporters has to meet continuously improving strict quality, legal, and ethical criteria for export-compliant manufacturing. Global health regulators continuously revise their levels, with successful exports growing constantly faster with every passing year [12]. In export destinations, non-tariff steps like safety and quality standards, and extensive documentation are mandatory requirements [12]. Further, the bioequivalence studies to establish the similarity of a particular drug with the conditions for sale and use in other countries having SRAs, is required [12].

As stated above, literature cites different kinds of hurdles that impede the growth of the pharmaceutical industry but none has studied the detailed reasons for the continuous lack of rectification of these hurdles. Further, no study emphasized exclusively medicine exports. Hence this study aimed to investigate the key factors, as opined by industrial experts from the domestic pharmaceuticals, as being profoundly impacting stakeholders [16] that are responsible for restricted export from the local pharmaceuticals especially to countries with SRA, along with the perceived reasons behind each hurdle and the proposed redressal. Though this study has been carried out in Pakistan yet its findings are envisioned to implicate in the developing countries globally.

## 2 Materials and methods

### 2.1 Interview schema. The study tools

A semi-structured interview guide, focusing on the questions that answer the research problem and gaps in the literature was developed based on an in-depth literature review [15, 17–19] and the current prevalent practices in the study setting. To develop the questionnaire, the services from two industrial experts were taken [17, 20]. The developed interview guide was subjected to cumulative and argumentative validation and later reliability was determined and made available for pilot testing for the thoroughness and relevance to the problems stated [21]. The interview guide was tested on two respondents from the pharmaceutical sector. Nevertheless, these results were not included in the final study. Afterward, slight changes in the guide were made before making it available for the real-time data collection (S1 File). The questions included the current status of the pharmaceutical industry, the status of local pharmaceutical R&D, barriers to, the perceived hurdles from regulatory and government, and their redressal. A question on the importance of bioequivalence study was asked to assess the industry’s willingness to invest, particularly for R&D activity and increase of exports. Questions were also included to investigate the profile of the participating companies in terms of the status of export, level of investments, resources allocation, and R&D, as perceived by industrial experts, and the status of ISO certification and location of the industry, i.e., within the factory area or otherwise to link the export barriers to the above parameters. The question on location was asked to assess the compliance with the regulator’s guidelines.

### 2.2 Methodology

#### 2.2.1 Ethical approval and consent to participate

A prior ethical approval was acquired from the research ethical committee, University of the Punjab, Lahore (Ref. D/30/FIMS) to conduct interviews for this study. A signed written informed consent was obtained from all participants. The identity of the respondents was protected by using respondent numbers (R). The respondents were free not to respond to any question, if they wanted.

#### 2.2.2 Study setting

The study was conducted on the pharmaceutical industry’s experts in Lahore, Karachi, Peshawar, and Quetta. The above cities are the provincial capitals of Punjab, Sindh, Khyber Pakhtun Khwa, and Baluchistan. Face-to-face interviews were conducted at the company’s invited premises in Lahore, while for the other cities, Zoom interviews were conducted.

#### 2.2.3 Study design

An exploratory qualitative study design [22] was employed using the purposive sampling technique [23, 24], initially to contact the industrial experts and later, snowball sampling [25] was used to reach the experts as referred by the previous interviewees.

#### 2.2.4 Study participants and inclusion criteria

The study samples comprised the industrial experts, expected to have knowledge and experience of pharmaceutical quality management, export, regulatory affairs, and marketing activities. Minimum experience and designation, respectively were 10 years and level of manager or above. The participants were expected to provide a neutral perspective on the questions asked. Before starting the study, the first author brainstormed and enlisted the names of the participants in the last week of February 2023. The recruitment process was started after the grant of ethical approval from 6^th^ March 2023 to 30^th^ March 2023.

#### 2.2.5 Sample size

The sample size was determined by applying the saturation point criteria used for qualitative study [26].

#### 2.2.6 Data collection

Face-to-face interviews of the participants were conducted from 1st April 2023 to 10th May 2023 for data collection by author 1, who is a female and had previously been trained in qualitative research, based on the guide prepared and accessed simultaneously. The said author has a degree in M. Phil, Pharmaceutics, and a working pharmacist for 12 years. All interviews were conducted in English. The audio was recorded with the consent of the participants. Details of the pharmaceutical companies from where the experts were included were noted. Companies were selected irrespective of their participation in exports. During interviews, to avoid bias, the modification, if any in the previously response followed by the next question was not considered [27].

#### 2.2.7 Data analysis

The data were analyzed using the thematic content analysis (TCA) [28]. Together, these insights have been described to help explain the main hurdles faced by the national pharmaceutical industry in targeting their export products to countries with SRAs and their business growth and remediations. First, all the audios were transcribed (in English) on paper to get familiarized with the data findings and generate initial notes. Secondly, a code was assigned to a single sentence, several sentences, or larger segments to summarize the actual sense of phenomenon described by the interviewee and relevant to answering the research questions. The co-researchers, in this study cross-checked the codes to ensure the credibility and trustworthiness of the data, as reported [29]. The list of codes was organized in MS Word to help identify connections among them. Codes describing similar phenomena were grouped under the same themes. Finally, the main findings were presented as the identified themes, and supporting quotations were also mentioned.

## 3 Results

**Fig 1:**
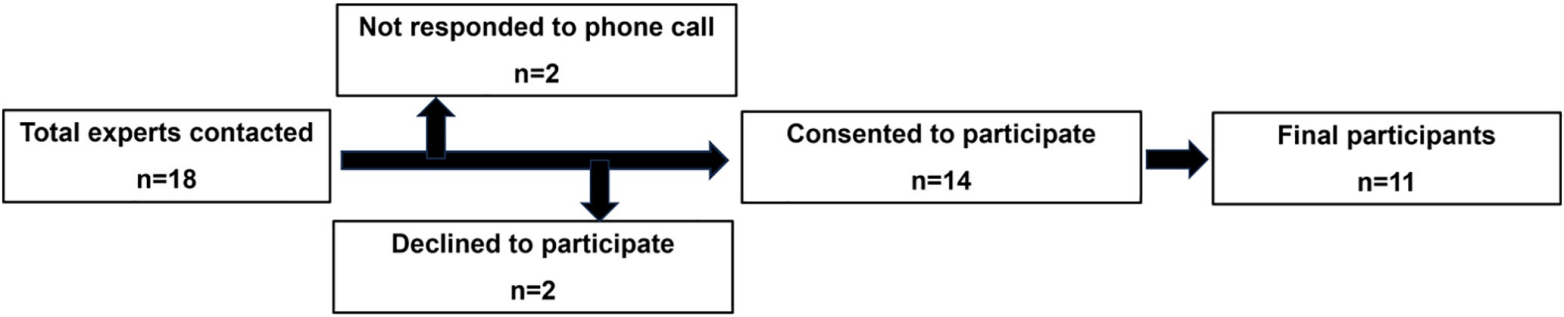
Attrition of the participants for study.

Eighteen (18) industrial experts were initially contacted, while 14 consented to participate (90% response rate). Among the remaining, 2 refused to participate apparently due to their busy schedules and one did not pick up the call (Fig 1). All participants (demographic details in Table 1) allowed audio recording. Saturation was achieved at the 9th interview and two additional interviews were conducted to confirm the saturation in emerging data, as reported [26]. The average interview duration was 28 min. Out of 11 participants, 10 were males and one female. Three participants were chemists having MS/M.Phil Analytical Chemistry degree while of the remaining eight, 6 experts had masters in Pharmacy (2, M.Phil Pharmaceutical Chemistry and 4, M.Phil Pharmaceutics) while 2 were PhD in Pharmaceutics. The participants’ minimum experience was ≥ 10 years. The age of the respondents varied from 35 to 55 years, as shown in Table 1.

**Table 1:** Relevant demographic details of the respondents (n = 11).

**Characteristics**	**Frequency**	**Characteristics**	**Frequency**		
**Location**		**Age**			
**Lahore**	6	35–45	6		
Karachi	2				
Peshawar	2	45–55	5		
Quetta	1				
**Gender**		**Experience**			
Male	10	10–12years	5		
Female	1	15–20years	6		
**Qualification**					
MS/M.Phil Analytical Chemistry	3	M.Phil pharmaceutics	4	PhD Pharmaceutics	2
		M.Phil Pharmaceutical Chemistry	2		
**Posts working**					
Manager QC	4	General manager	3		
Manager regulatory affairs	3	QA manager	1		

The industrial experts were reluctant to respond to the questions related to the investments and total area covered by the factory, stating that this was not in their scope. Among the chosen companies of experts 5, out of 11 were ISO-certified for quality manufacturing, 3 of 11 were located in the factory area, none (0) were reported to have the R&D on new molecules, 2 out of 11 were in contract-manufacturing of innovators from the foreign companies, while 2 were even without any R&D section. The remaining 7 companies had product development in the R&D section. Only 1 firm has shown a capability to export to countries with SRA at present as it is approved by MHRA-UK. Characteristics of the chosen pharmaceutical industry are given in Table 2.

**Table 2:** Characteristics of the selected industry.

Characteristics	Frequency	Characteristics	Frequency
**Location**			
Industrial area	3	Nonindustrial area	8
**ISO Certified**		**Involved in export**	
ISO Certified	5	Exporting	6
Non-ISO certified	6	Not exporting	5
**Export to SRA**		1	
**Research and development**			
Generic manufacturing based on R&D	7	No R&D	2
Innovators on contract	2	New products	0

### 3.1 Thematic content analysis

The analysis of the acquired data yielded 4 themes with 16 codes representing about the industrial perspectives on pharmaceutical export and growth. The emerging themes, categories/codes, and supporting quotations are highlighted in Table 3. The following themes were identified during the analysis.

**Table 3:** Identified themes, categories/codes, and supporting quotations.

Theme	Respondent	Supporting quotations (selected)
**Theme 1: Quality and Compliance Concerns**		
Lesser use of the latest technology in the industry	R1–R11	R4: In small domestic industries, there is less use of the latest technology. There is no concept of continuous manufacturing, rather industry fills up the manufacturing needs only. The big companies with quality standards use the latest technology.
Lack of compliance with cGMPs		R2: GMP guidelines and standards are the same all over the world but only a few domestic companies follow these guidelines. The GMP implementation is subtle.
Working standards are being used for quality control analysis		In the local industry, the working standards are employed for the drugs analysis which is a challenge for the quality of pharmaceutical products. DRAP should make a chemical resource from authentic sources for the primary standards to be provided at subsidized rates.
**Theme 2: Lack of Research and Development (R&D)**		
Pharmaceutical manufacturing is almost 100% generics	R1–R11	R3, R4, and R5: There is complete lack of innovative product development. Only the generics are being manufactured in the domestic pharmaceutical industry.
No academia-industry linkage (AIL) for basic and applied research		R2, R6, and R7: The academia-industry linkage (AIL) for R&D is scarce, relative to the developed countries where industry-related research is done in the academic institutes to be applied in industry.
No utilization of central research fund		R1, R3, and R4: The pharmaceutical industry submits a fund to DRAP at a rate of 1% of their total annual sale, which is deposited as a huge amount and so far, there is no use of that fund
**Theme 3: Major hurdles faced by local industry in export and growth**		
SRA regulatory compliance	R1–R11	R3 and R4: Compliance with SRAs is the biggest challenge, the local industry is confronted with.
		R3: There is not a single FDA-approved plant. Only one industrial unit located in Lahore is MHRA-UK-approved.
Price of the products		R4: The cost of manufacturing complying with GMP standards is the biggest challenge for the industry. Instead of subsidy, the industry needs only support in terms of process expedition of export documentation from DRAP.
Regulatory hurdles		R7: Cooperation of DRAP is necessary to grow professionally and make some standing of the local pharmaceutical companies at the international level.
		R1 and R2: DRAP should provide pieces of training on export enhancement and documentation should be expedited to facilitate the export.
No requirement for bioequivalence studies for local registration		R4 and R5. R6: Bioequivalence studies should be set mandatory for the registration of every generic by DRAP.
		R4: The experts for the performance of bioequivalence studies are unavailable and none of the centers is of a level, acceptable by SRAs.
Industrial dependence on imported raw materials		R2, R3, and R7: The local industry depends almost 100% on imported raw materials, a major challenge in export. DRAP should facilitate and make the import of raw materials a simple procedure with lesser time consumption. DRAP should have a list of multiple approved raw material vendors for raw material import and exempt on-port raw material clearance by assistant drug controllers (ADCs) to avoid delay in their receiving.
**Theme 4: Ways to improve pharmaceuticals export**		
Improving the market perception of the domestic industry	R1–R11	R4, R5, and R8: Improving the image of the local industry in the international market is a main tool through which exports can be enhanced. Better marketing strategies and combined efforts of industry and government through private-public partnerships can help in improving the image of local industry internationally.
Improve infrastructure and technology		R3, R6, and R9: Better infrastructure of the local industry and use of the latest technology and research-based formulations can enhance the export.
Complying with SRAs.		R2 and R3: Developing the local industry to meet SRA standards can enhance exports to developed as well as underdeveloped countries.
		R4 and R5: DRAP should get approval from the Pharmaceutical Inspection Co-operation Scheme (PICS) for a better regulatory image.
Skill development and workforce		R3 and R6: Provision of a better pool of skilled workers in industries by government cooperation can improve the quality of products and hence the export.
Price deregulation		R2 and R6: The price of the medicines should be deregulated so that the industry could get a better return on investment and invest more in research and provide quality products in the market.
		R4: Price allocation for the products should be rational, depending on the quality of manufacturing.

#### 3.1.1 Theme 1: Quality and compliance concerns

Related to the present status of the domestic pharmaceutical industry, in comparison to the developed countries all the respondent raised quality and compliance issues. Two respondents indicated the absence of a concept of continuous manufacturing in local pharmaceuticals which reflected the reluctance of the domestic industry to improve the quality manufacturing standards. According to them, pharmaceutical industry just fulfilled the manufacturing needs. One participant from a well-reputed and high-quality company believed that the quality standards were the same in all companies, the difference was made only by a compliance with cGMP guidelines which was only followed by a few companies. Another main concern raised by the industrial expert was the use of working standards in quality control testing of the active pharmaceutical ingredients (APIs), rather than the primary or secondary reference standards. According to them, this practice caused a reduced reliability of the quality control testing and in turn, the product image.

#### 3.1.2 Theme 2: Lack of research and development

The study participants’ responses showed that there was almost zero R&D in the national pharmaceutical industry as compared to that in the developed world where innovators were being prepared. The focus of the domestic industry was only the generic manufacturing with limited research. Industrial profile data also revealed that some companies even did not have R&D sections. Lack of R&D showed that the industry did not intend to invest in R&D to bring new and competitive products to the global market. Lack of R&D could be associated with the absence of academia-industry linkage (AIL) as agreed by the respondents. Respondents also said that there was no utilization of the central research fund (CRF) deposited to the DRAP by the pharmaceutical industry at a rate of 1% of their gross annual sale before tax which was a huge money collected with DRAP and the Ministry of Health and was of no use so far.

#### 3.1.3 Theme 3: Major hurdles faced by local industry in export and growth

Most of the industrial experts opined that compliance with SRAs was the biggest hurdle for pharmaceutical export to the SRA countries. Locally the complex and lengthy regulatory processes did not facilitate the export of pharmaceuticals. Delays in obtaining necessary approvals and licenses could impact the timely entry of domestic pharmaceutical products into the international markets. Another major reason highlighted was the dependency of the pharmaceutical industry on imported raw materials. Disruptions in the supply chain, either due to logistical issues or the shortage of raw materials supply, could impact the timely production and delivery of pharmaceutical products. A strict DRAP regulation on the prices of pharmaceutical products was taken as one of the major hurdles to export. Respondents also highlighted that the local pharmaceutical industry was mainly involved in the production of generics which are required to be bioequivalent to their respective innovators for approval in countries with SRAs. While it was not a mandatory regulatory requirement in Pakistan, might be due to the lack of facilities for bioequivalence testing and if there were any, no experts on bioequivalence are available there. This was another hurdle reported by the industrialists for the growth of the local pharmaceutical industry.

#### 3.1.4 Theme 4: Ways to improve export from local industry

Positive market perception and image of the locally manufactured products was highly essential for enhancing the export of the local pharmaceutical industry as highlighted by the respondents. Access to modern manufacturing facilities and technologies was essential for producing high-quality pharmaceuticals and improving exports. A lack of investment in infrastructure and technology could limit the industry’s ability to scale production and meet international demands. Reliability in the supply chain was essential for building trust with the international partners. The experts said that the national pharmaceutical industry should try to comply with the SRAs to improve its place in the markets and gain a share of exports. The requirement of a skilled workforce was also stated as crucial for pharmaceutical manufacturing by industrial experts. The availability of skilled personnel, both in terms of technical expertise and knowledge of regulatory compliance, could facilitate the industry’s ability to meet international standards. One respondent viewed that the price of pharmaceutical products should be kept open (deregulated) and should not be controlled by the regulatory authority to compensate the expense of the industry and, in return, the industry could produce better quality medicines.

## 4 Discussion

The present study was focused on finding the reasons, and the redressals of the barriers and gaps for the pharmaceutical export, as the industrial experts perceive. The trigger for this study was the perception obtained from the pharmacy academic experts, another impactful stakeholder [30, 31]. The literature has provided limited insight from the industrial perspective on the issues related to pharmaceutical export, growth, and the current status of the domestic industry [15]. Some industrial respondents indicated domestic pharmaceutical industry was comparable to that of the industries in developed countries. Yet few thought that comparisons could not be made as the industry composes small-, medium- and big-sized companies. According to them, only the big companies were comparable. The pharmaceutical companies could be categorized as big-, medium- or small-sized based on their investment, production capacity, and market share [32] Nevertheless, clues could not be found from the regulatory legislation to support such industrial classification. Since it had been declared to the study participants that the responding, or otherwise to a question was their discretion, the respondents were reluctant to respond on the investment of a company and area covered by the factory. Further, this study was based on the perception of the respondents, therefore actual data on the production of the companies and the market share were out of scope for this study.

The major issues for the pharmaceutical export highlighted by the study participants were the complete or partial lack of compliance with the cGMP standards and scarcity of the high-tech equipment. The above was in line with the literature reporting use the of non-GMP, ICH- and FDA-guidelines-compliant equipment [33].

The country has a drug price regulation system, where DRAP assigns/decides a price for a product in response to an application from a company [16, 34]. The majority of the industrial experts opined irrationally lower price given, for the domestic pharmaceutical product by DRAP, was a cause of lower profit which was not enough to allow industry for further investments on meeting cGMP, R&D or higher instrumental requirements. The above findings were in line with the literature ascribing the financial limitation besides technological, infrastructural administrative shortcomings of the domestic industry [17]. Furthermore, the use of traditional production approaches, rather than continuous manufacturing and a limited utilization of the latest technology in small-scale pharmaceutical companies have been reported to hamper the efficiency and ability to meet the global quality standards and hinder competitiveness in international markets [35]. The industrial experts mentioned that import of APIs was not easy and the cost of the primary and secondary standards made their local availability difficult. They opined that they should be purchased by DRAP and supplied to the domestic industry through DRAP-approved dealers for convenience. However, inquiring from the regulators revealed that both are not the DRAP’s mandates. Nevertheless, the import of APIs and the availability of primary and secondary standards have been declared as the barriers to quality manufacturing, thus the DRAP and government should take measures to resolve the issues.

The countries with SRAs, enforce the bioequivalence (BE) testing and certification from the qualified agencies [36]. However, prior BE testing for domestic generics has not been a regulatory requirement for registration, further, the country does not have bioequivalence (BE) centers and experts in the field. Consequently, a company that intends to conduct bioequivalence testing of its generic, is compelled to get this investigation from abroad, which incurs substantial expenses and involves foreign exchange and money transfer processes which further complicate the situation [37]. In line with the above, the generics export to the developed countries with SRA was identified by the study participants as challenging. Recently, 6 bioequivalence centers, licensed by the DRAP, inclusive of a provincial government-owned facility, the Pakistan Drug Testing & Research Center in Lahore have been established and are also being expanding in number [38]. However, none of these centers hold accreditation from international bodies. Their performance in facilitating exports and their compliance with regulatory requirements for generic drug registration or international standards remain uncertain. It’s worth noting that two BE centers were previously discontinued, possibly due to a lack of clients, reflecting the unwillingness of the pharmaceutical companies the conduct BE testing. The study participant perceived the above as the regulator gap and framing a policy by DRAP for BE testing and facilitation of their respective international accreditation was mandatory for the promotion of export. Moreover, the BE expert could be pooled from the academic institutes under AIL programs.

Lack of R&D was reported as another barrier that was responsible for the stunted growth of the domestic pharmaceutical industry. The R&D is a significant activity in the pharmaceutical sector, wherein global pharmaceutical companies has dedicated a significant amount of $141 billion annually to R&D endeavors [39]. Exploration of new chemical entities (NCEs) is an extensive and expensive undertaking but typically spans about 12–13 years from synthesis to a new product and market availability after its successful passing all the trials. In addition to the duration, the expenses involved (about a staggering $2,558 million) are substantial for the development of an NCFs [40]. Furthermore, without the research and development efforts in the pharmaceutical industry, the world would not have witnessed the advancements in medicines that have ultimately saved millions of lives globally [41]. Despite the lengthy time and immense cost, only 2 out of every 10,000 substances survive through all the phases of trials and development to eventually become a market-ready product [42]. The domestic firms continue to rely on research conducted in other countries, and the accessibility of high-quality drugs remains a challenge [5]. At the same time, the local industry encounters the formidable challenge of bringing a new drug to market without the help of the stakeholders. Nonetheless, pharmaceutical companies are willing to embrace this risk in anticipation of reaping rewards in the future.

Nevertheless, for a country like Pakistan, the potential economic advantages of investing in research for new drugs could be significant. It has the potential to result in substantial savings, amounting to money annually, in healthcare expenditures by introducing innovative and more effective medications, ultimately saving millions of lives globally.

In reply to the question asked on utilization of CRF by DRAP the industrial experts viewed there is zero utilization so far while a huge amount was collected with DRAP. The Drug Act 1976, since its enactment mandates pharmaceutical companies to allocate 1% of their gross profits to the government to conduct. There is no data available to demonstrate the total amount collected and utilized over time and what impact it has had on the drug-related domestic R&D [5]. There were some unrevealed aspects, yet the literature has cited as barriers to export, such as the tax system in the country. The Normal Tax Regime (NTR) applies to the sale of domestically produced medications, while the import of finished drugs incurs an income Tax under the Final Tax Regime (FTR) [43].

For ways to enhance pharmaceutical export, the industrial experts opined that the market perception about domestically manufactured pharmaceutical products was warranted. As global pharmaceutical market is highly competitive, with established players from various countries. Competing with well-established manufacturers requires a strong marketing and distribution strategy, as well as competitive pricing. Struggling of domestic pharmaceutical companies to compete on the above fronts could positively influence the export potentials. In the recent past, local media reported incidents involving counterfeit medicines. One of the most significant cases occurred at the Punjab Institute of Cardiology-Lahore, where reportedly adulterated and counterfeit medicines had led to the tragic deaths of around 100 cardiac patients [44]. A report titled "Why our drug markets askew," published in the daily DAWN, claimed a significant proportion of domestic drugs as either fake or counterfeit [18]. However, though domestic industry is majorly manufacturing the generics but, the pharmaceutical companies follow the cGMP standards and are comparable to that of the other developed countries. Regarding fulfilling the SRA regulatory requirements and standards, the respondents regarded it difficult. At present there is only one domestic plant that is accredited to MHRA-UK and can export and sell products in the UK [12]. The respondents linked the lack of accreditation to the strict price control policy of DRAP, like the lack of use of high-tech cGMP-compliant instruments and respective standards guidelines, as stated earlier. It was narrated that the DRAP only wanted quality manufacturing from the industry without considering the expenditures on it. Linking the export barrier to price indicated that the domestic product prices were irrational. Findings triggered a view on price regulation. The drug prices based on the actual industrial inspection and extent (percentage) of cGMP compliance, is a rational system of devising the prices. In this way, companies with greater investment and intention to produce quality could be identified and compensated in terms of adopting the latest technology and methods. This would boost the industrial confidence and in turn, business and export.

The price deregulation of the locally manufactured medicines was perceived by one of the respondents to increase the product quality, and thus their export. Nevertheless, the above opinion of the industrial expert was contravening the WHO guidelines on pricing and the DRAP price control policy over exports. Indeed, DRAP exempts the local products approved for export to developed countries (like the USA, UK, EU countries, Japan, Australia, or WHO) from price control in the local market to encourage manufacturing and export of quality drugs subject to the local FOB price for export not less than the ex-factory price [34]. Thus, linking the DRAP-regulated price system as a hurdle is not rational.

Industrial experts opined that the improvement of the infrastructure and the adoption of the latest technology can enhance manufacturing quality and this will in turn increase exports. The data on the industrial profile indicates that most of the companies were not even present in the factory area which is a contradiction to the DRAP requirement which requires the factory should not be located in any residential or commercial area. Pharmaceutical export depends on the innovation and development of new drugs or formulations. Data show that few companies lack an R&D department even for product development which is also a noncompliance to the manufacturing standards accepted internationally. There is a need to incorporate skilled industrial professionals in the domestic pharmaceutical industry and DRAP. DRAP was established to recruit and nurture skilled professionals, modernize the regulatory system, establish pharmacovigilance practices, upgrade drug testing laboratories, and improve human resources and equipment in the pharmaceutical sector [45–47]. DRAP should meet the above aim and should focus on getting certification from PICS and WHO to increase access to bigger stringent markets [45].

Respondents also took part in some informal discussions that revealed some out-of-context facts about the industrial intention for investment. Some experts stated that the export could not be achieved without accomplishing SRA requirements. Some respondents mentioned that the company owners just intend to generate revenue by producing medicines fulfilling the needs of the low-income local population. The companies are earning handsome revenue from the local supply of medicine while the export on the other hand demands activities and bigger investments and that does not come under their agenda.

## 5 Limitations of the study

Such types of studies, as the current one are usually limited to the personal design biases. However, in this study, the respondents were encouraged to express themselves without any interruption. The probing questions were asked only to clarify the aspects under discussion without any additions by the interviewer. Further, no alteration in response was considered in coding if any respondent changed one’s previous response based on the next question, as indicated previously. The participants included in this study were mainly from Lahore due to convenience. A further limitation of such studies is no or partial knowledge and wrong or misleading perception for any question. For instance, a respondent of this study seemed to be unfamiliar with the pricing system of medicines and the relevant guidelines and local policies. This issue could be addressed using a correct fact against a misleading response, where applicable. The sample size was confined to only 11 experts this could be another limitation and, in the future, the study can be expanded with a greater number of participants, particularly MNCs from Karachi, the main business hub of Pakistan. Moreover, further research is necessary to provide a comprehensive understanding of the problem and explore the perspectives of drug regulators, who are also a significant stakeholder of pharmaceutical industry [16]. Nevertheless, such shortcomings are inherent in these types of studies, though, the findings could be generalizable with caution [48].

## 6 Conclusion

The domestic pharmaceutical industry is a market of generics and has export potential. Although industry, and thus the products are comparable to that of the developed countries. Certain highlighted barriers impeding the invasion of domestic pharmaceutical products to the strictly regulated authority were: difficulty in compliance with the requirements of strictly regulatory authorities, partial or complete lack of research and development, generic manufacturing without bioavailability studies, price regulation by regulatory authority without considering the expenditures on GMP-compliant industrial working, delayed approval procedures of regulatory authority, strict procedures adopted by the SRA countries, such as certification of the local pharmaceuticals with SRA authorities, which is costlier for the industry and lack of bioequivalence test centers acceptable by strictly regulated authorities. Certain measures, that could help to readdress the above issues are: developing research and development for the growth of the pharmaceutical sector, academia-industrial collaboration, with regulatory assistance for enhanced research and development, facilitation in bioequivalence study, and setting it as a mandatory regulatory step for approval of generic manufacturing, removal of difficulties in importing of APIs, and making reference standards available. Further, the image of the pharmaceutical industry should be enhanced by DRAP through the representation of the national industry at international forums by combined academia-industry programs and positive marketing strategies.

## 7 Recommendations

### 7.1 Recommendations for industry

- The industry should adopt the latest technology and show total compliance with the cGMPs and ICH guidelines for the production of better-quality products.
- The industry should hire skilled personnel with professional backgrounds who are skilled in handling manufacturing and quality-related issues,
- Industry should strive to achieve accreditations with ISO for a better image.
- Industry should launch the independent R&D sections, in collaboration with academia.

### 7.2 Recommendations for DRAP

- DRAP should ensure compliance of industrial manufacturing to the latest standards
- DRAP should issue NOCs only to those premises which are located in industrial areas for the long-term growth of industry and to avoid exposure of the population to the wastes of the industry.
- DRAP should get the PICS membership to represent the country with good regulatory control to improve the image of national pharmaceuticals.
- Government support to DRAP in terms of surprise audits and visits to the industry can help to ensure compliance with the set standards.

### 7.3 Recommendations for academia

Academia-industry linkage can be an opportunity for the interinstitutional mobility of experts and improved research, mobility of experts industrial-relevant research.

## Supporting information

S1 File (DOCX)

## Abbreviations

DRAP: Drug Regulatory Authority of Pakistan
MHRA-UK: Medicines and Healthcare Products Regulatory Agency- United Kingdom
PICS: Pharmaceutical Inspection Cooperation Scheme
QC: Quality control
R&D: Research and Development
SRA: Stringent regulatory authorities
US-FDA: United States Food and Drug Administration
