# Supplementary material for: Industrial prospects on regulatory gaps and barriers in pharmaceutical exports and their counteraction: Local experiential with global implication
Source: PLoS One. 2024 Jul 19;19(7):e0305989. doi: 10.1371/journal.pone.0305989 (PMC11259304; doi:10.1371/journal.pone.0305989)
Supplement: S1 File — (DOCX) [file pone.0305989.s001.docx]

**Interview Guide**

(Industrial Part)

We want to highlight the issues; the pharmaceutical industry is facing for operation of Export??

This questionnaire will be used only for research purpose and no information will be published or shared anywhere with name of Company or the person.

You may choose not to respond any question.

| **1** | **Code of Company** |  |
| --- | --- | --- |
| **2** | **Location** | **[ ] Industrial zone**  **[ ] other** |
| **3** | **Type of Company** | **[ ]National**  **[ ] Multinational** |
| **4** | **Designation of the Person being interviewed** |  |
| **5** | **Starting year** |  |
| **6** | **Number of the manufactured products** |  |
| **7** | **Land of Factory x Total covered area** |  |

1. How do you see the Pakistani Pharmaceutical Industry compared to the Pharma Industry in the developed countries including SRA countries?

----------------------------------------------------------------------------------------------------------------------------------------------------------------------------------------------------------------------------------

1. Is there a dedicated Research & Development department in this company?

----------------------------------------------------------------------------------------------------------------------------------------------------------------------------------------------------------------------------------

1. What is the estimated investment in the R & D, Equipment and Utilities, etc.?

----------------------------------------------------------------------------------------------------------------------------------------------------------------------------------------------------------------------------------

1. Is there any legal requirement for the import of APIs to conduct stability studies before submission of the registration dossier?

----------------------------------------------------------------------------------------------------------------------------------------------------------------------------------------------------------------------------------

1. How do you maintain a continuous electric supply in your plant? Power backup etc.

----------------------------------------------------------------------------------------------------------------------------------------------------------------------------------------------------------------------------------

1. What are the main products being manufactured and the main sections in the Company?

--------------------------------------------------------------------------------------------------------------------------------------------------------------------------------------------------------------------------------------------

1. Does this Company already have any certification on quality standards? OR has this company applied for any international certificate?

----------------------------------------------------------------------------------------------------------------------------------------------------------------------------------------------------------------------------------------------

1. Is Quality control testing of the products being done as per the official Compendium (USP/BP)?

----------------------------------------------------------------------------------------------------------------------------------------------------------------------------------------------------------------------------------------------

1. What is the minimum qualification & and experience of the technical staff involved in:

[A] Production --------------------------------------

[B] Quality Control --------------------------------

[C] Quality Assurance -----------------------------

1. How does Industry determine the training needs of the staff and how are they trained (Local/foreign)?

--------------------------------------------------------------------------------------------------------------------------------------------------------------------------------------------------------------------------------------------

1. Do regulatory authorities help in training the staff or provide any training to the industrial staff for continuous learning?

----------------------------------------------------------------------------------------------------------------------------------------------------------------------------------------------------------------------------------

1. Does this Company export its Products? Or does this Company have a plan of export?

--------------------------------------------------------------------------------------------------------------------------------------------------------------------------------------------------------------------------------------------

1. Have this Company ever been inspected by a foreign regulatory authority? What was the purpose?

--------------------------------------------------------------------------------------------------------------------------------------------------------------------------------------------------------------------------------------------

1. What are the biggest challenges for export that the Pharma sector is confronted with?

--------------------------------------------------------------------------------------------------------------------------------------------------------------------------------------------------------------------------------------------

1. Does this company perform vendor qualification for the procurement of Materials?

--------------------------------------------------------------------------------------------------------------------------------------------------------------------------------------------------------------------------------------------

1. Does this company have the latest GMP model equipment?

--------------------------------------------------------------------------------------------------------------------------------------------------------------------------------------------------------------------------------------------

1. What are your views on the bio-equivalence of local generic products?

--------------------------------------------------------------------------------------------------------------------------------------------------------------------------------------------------------------------------------------------

1. Do you think bio-equivalence studies will improve the image of local products which can increase the export potential?

-------------------------------------------------------------------------------------------------------------------------------------------------------------------------------------------------------------------------------------------

1. What are the hurdles in bio-equivalence studies in local Industry?

--------------------------------------------------------------------------------------------------------------------------------------------------------------------------------------------------------------------------------------------

1. What are the requirements for meeting international standards and favoring export?

--------------------------------------------------------------------------------------------------------------------------------------------------------------------------------------------------------------------------------------------

1. Is regulatory legislation required in improving local production matching the international standards?

--------------------------------------------------------------------------------------------------------------------------------------------------------------------------------------------------------------------------------------------

1. What role Ministry of commerce can play for contribution of local industry in national economy?

--------------------------------------------------------------------------------------------------------------------------------------------------------------------------------------------------------------------------------------------

1. What is the value of local Research on the time-tested drugs done in Academia?

------------------------------------------------------------------------------------------------------------------------------------------------------------------------------------------------------------------------------------------

1. What are your views about an export start-up program? What role can you play in the export start-up program?

--------------------------------------------------------------------------------------------------------------------------------------------------------------------------------------------------------------------------------------------

1. What can be the effective utility of the fund submitted to DRAP from the Pharmaceutical industry in lieu of research and development?

----------------------------------------------------------------------------------------------------------------------- ------------------------------------------------------------------------------------------------

1. What is a proposal to boost pharmaceutical exports? What role DRAP can play in it?

--------------------------------------------------------------------------------------------------------------------------------------------------------------------------------------------------------------------------------------------
